# A Weighted Combination Method for Conflicting Evidence in Multi-Sensor Data Fusion

**DOI:** 10.3390/s18051487

**Published:** 2018-05-09

**Authors:** Fuyuan Xiao, Bowen Qin

**Affiliations:** School of Computer and Information Science, Southwest University, No.2 Tiansheng Road, BeiBei District, Chongqing 400715, China; qinbowen_swu@163.com

**Keywords:** multi-sensor data fusion, conflicting evidence, Dempster–Shafer evidence theory, belief entropy, similarity measure, data classification, fault diagnosis

## Abstract

Dempster–Shafer evidence theory is widely applied in various fields related to information fusion. However, how to avoid the counter-intuitive results is an open issue when combining highly conflicting pieces of evidence. In order to handle such a problem, a weighted combination method for conflicting pieces of evidence in multi-sensor data fusion is proposed by considering both the interplay between the pieces of evidence and the impacts of the pieces of evidence themselves. First, the degree of credibility of the evidence is determined on the basis of the modified cosine similarity measure of basic probability assignment. Then, the degree of credibility of the evidence is adjusted by leveraging the belief entropy function to measure the information volume of the evidence. Finally, the final weight of each piece of evidence generated from the above steps is obtained and adopted to modify the bodies of evidence before using Dempster’s combination rule. A numerical example is provided to illustrate that the proposed method is reasonable and efficient in handling the conflicting pieces of evidence. In addition, applications in data classification and motor rotor fault diagnosis validate the practicability of the proposed method with better accuracy.

## 1. Introduction

Multi-sensor data fusion technology has received significant attention in a variety of fields, as it combines the collected information from multi-sensors, which can enhance the robustness and safety of a system. In wireless sensor networks applications, however, the data that are collected from the sensors are often imprecise and uncertain [1]. How to model and handle the uncertainty information is still an open issue. To address this problem, many mathematical approaches have been presented, such as the fuzzy sets theory [2,3], that focuses on the intuitive reasoning by taking into account human subjectivity and imprecision; the intuitionistic fuzzy sets theory [4] which generalizes fuzzy sets by considering the uncertainty in the assignment of membership degree known as the hesitation degree; evidence theory [5,6,7], as a general framework for reasoning with uncertainty, with understood connections to other frameworks such as probability, possibility, and imprecise probability theories; rough sets theory [8,9] where its methodology is concerned with the classification and analysis of imprecise, uncertain, or incomplete information and knowledge, which is considered one of the first non-statistical approaches in data analysis; evidential reasoning [10,11] which is a generic evidence-based multi-criteria decision analysis (MCDA) approach for dealing with problems having both quantitative and qualitative criteria under various uncertainties including ignorance and randomness; Z numbers [12,13], that intend to provide a basis for computation with numbers which are not totally reliable; D numbers theory [14,15,16,17] which is a generalization of Dempster–Shafer theory, but does not follow the commutative law; and so on [18,19,20,21]. In addition, mixed intelligent methods have been applied in decision making [22], risk analysis [23], supplier selection [24], pattern recognition [25], classification [26], human reliability analysis [27], and fault diagnosis [28], etc. In this paper, we focus on evidence theory to deal with the uncertain problem of multi-sensor data fusion.

Dempster–Shafer evidence theory was firstly presented by Dempster [5] in 1967; later, it was extended by Shafer [6] in 1976. Dempster–Shafer evidence theory is effective to model both of the uncertainty and imprecision without prior information, so it is widely applied in various fields for information fusion [29,30,31,32]. Nevertheless, it may result in counter-intuitive results when combining highly conflicting pieces of evidence [33]. To address this issue, many methods have been presented in recent years [34,35,36]. On the one hand, some researchers focused on amending Dempster’s combination rule. On the other hand, some researchers tried to pretreat the bodies of evidence before using Dempster’s combination rule. In terms of of amending Dempster’s combination rule, the major works contain Smets’s unnormalized combination rule [37], Dubois and Prade’s disjunctive combination rule [38], and Yager’s combination rule [39]. However, the modification of combination rule often breaks the good properties, like commutativity and associativity. Furthermore, if the sensor failure gives rise to the counter-intuitive results, the modification of combination rule is considered to be unreasonable. Therefore, in order to resolve the fusion problem of highly conflicting pieces of evidence, researchers prefer to pretreat the bodies of evidence. With respect to pretreating the bodies of evidence, the main works contain Murphy’s simple average approach of the bodies of evidence [40], and Deng et al.’s weighted average of the masses based on distance of evidence [41]. Deng et al.’s method [41] conquered the deficiency of the method in [40]. However, the impact of evidence itself was neglected in the decision-making process.

Hence, in this paper, a weighted combination method for conflicting pieces of evidence in multi-sensor data fusion is proposed to resolve fusion problem of highly conflicting evidence. First, the credibility degree of each piece of evidence is determined on the basis of the modified cosine similarity measure of basic probability assignment [42]. Then, credibility degree of each piece of evidence is modified by adopting the belief entropy function [43] to measure the information volume of the evidence. Finally, the modified credibility degree of each piece of evidence is used to adjust its corresponding body of evidence to obtain the weighted averaging evidence before using Dempster’s combination rule. A numerical example is given to illustrate the feasibility and effectiveness of the proposed method. Additionally, the proposed method is applied in data classification and motor rotor fault diagnosis, which validates the practicability of it.

The rest of this paper is organized as follows. Section 2 briefly introduces the preliminaries of this paper. After that, Section 3 proposes the novel method, which is based on the similarity measure of evidence and belief function entropy. Then, Section 4 gives a numerical example to show the effectiveness of the proposed method. A statistical experiment is carried out in Section 5. Afterwards, the proposed method is applied to Iris data set classification, and motor rotor fault diagnosis is performed in Section 6. Finally, Section 7 gives the conclusions.

## 2. Preliminaries

### 2.1. Data Fusion

Data fusion can be identified as a combination of multiple sources to obtain improved information with less expensive, higher quality, or more relevant information [44]. General data fusion structure can be classified into three types based on the different stages: data-level, feature-level, and decision-level, as referred in [45].

In the data-level fusion, all raw data from sensors for a measured object are combined directly. Then, a feature vector is extracted from the fused data. Fusion of data at this level consists of the maximum information so that it can generate good results. However, sensors used in the data-level fusion, such as the sensors reporting vibration signals, must be homogeneous. As a consequence, the data-level fusion is limited in the actual application environment, because many physical quantities can be measured for a more comprehensive analysis. In the feature-level fusion, heterogeneous sensors can be used to report the data. According to the types of collected raw data, the features are extracted from the sensors. Then, these heterogeneous sensor data are combined at the feature-level stage. All of the feature vectors are combined into a single feature vector, which is then utilized in a special classification model for decision-making. In the decision-level fusion, the processes of feature extraction and pattern recognition are sequentially conducted for the data collected from each sensor. Then, the produced decision vectors are combined by using decision-level fusion techniques such as the Bayesian method, Dempster–Shafer evidence theory, or behavior knowledge space.

Because of the advantages of multi-sensor data fusion technology, it has been widely applied in various fields, such as in fault diagnosis [46,47,48], target tracking [49,50], health care analysis [51,52], image processing [53], attack detection [54], estimation of ship dynamics [55], and characterization of built environments [56].

In this paper, we focus on decision-level fusion, and try to improve the performance of the system based on Dempster–Shafer evidence theory.

### 2.2. Dempster-Shafer Evidence Theory

Dempster–Shafer evidence theory was firstly proposed by Dempster [5] and was then further developed by Shafer [6]. Dempster–Shafer evidence theory, as a generalization of Bayesian inference, asks for weaker conditions, which makes it more flexible and effective to model both the uncertainty and imprecision. The basic concepts are introduced as below.

**Definition** **1.** *Let U be a set of mutually exclusive and collectively exhaustive events, indicated by*

(1) $$U=\{C_{1},C_{2},\ldots ,C_{i},\ldots ,C_{N}\}.$$

*The set U is called frame of discernment. The power set of U is indicated by $2^{U}$, where*

(2) $$\begin{array}{c} 2^{U}=\left\{\emptyset ,\left\{C_{1}\right\},\left\{C_{2}\right\},\ldots ,\left\{C_{N}\right\},\left\{C_{1},C_{2}\right\},\ldots ,\left\{C_{1},C_{2},\ldots ,C_{i}\right\},\ldots ,U\right\}, \end{array}$$

*and* ∅ *is an empty set. If $A\in 2^{U}$, A is called a proposition or hypothesis.*

**Definition** **2.** *For a frame of discernment U, a mass function is a mapping m from $2^{U}$ to [0, 1], formally defined by*

(3) $$m:2^{U}\to \left[0,1\right],$$

*which satisfies the following condition:*

(4) $$m\left(\emptyset \right)=0\;and\sum_{A\in 2^{U}}m\left(A\right)=1.$$

In Dempster–Shafer evidence theory, a mass function can be also called as a basic probability assignment (BPA). If m(A) is greater than 0, *A* will be called as a focal element, and the union of all of the focal elements is known as the core of the mass function.

**Definition** **3.** *For a proposition A⊆U, the belief function $Bel:2^{U}\to \left[0,1\right]$ is defined as*

(5) $$Bel\left(A\right)=\sum_{B\subseteq A}m\left(B\right).$$

*The plausibility function $Pl:2^{U}\to \left[0,1\right]$ is defined as*

(6) $$Pl\left(A\right)=1-Bel\left(\bar{A}\right)=\sum_{B\cap A\neq \emptyset }m\left(B\right),$$

*where $\bar{A}=U-A$.*

Apparently, Pl(A) is equal or greater than Bel(A), where the function Bel is the lower limit function of proposition *A* and the function Pl is the upper limit function of proposition *A*.

**Definition** **4.** *Let the two BPAs be $m_{1}$ and $m_{2}$ on the frame of discernment U. Assuming that these BPAs are independent, Dempster’s rule of combination, denoted by $m=m_{1}⊕m_{2}$, known as the orthogonal sum, is defined as below:*

(7) $$m\left(A\right)=\left\{\begin{array}{c} \begin{array}{cc} \frac{1}{1-K}\sum_{B\cap D=A}m_{1}\left(B\right)m_{2}\left(D\right), & \;A\neq \emptyset , \end{array}\\ \begin{array}{cc} 0, & \;A=\emptyset , \end{array} \end{array}\right.$$

*with*

(8) $$K=\sum_{B\cap D=\emptyset }m_{1}\left(B\right)m_{2}\left(D\right),$$

*where B and D are also the elements of $2^{U}$, and K is a constant that presents the conflict between the two BPAs.*

Note that Dempster’s combination rule is only practicable for the two BPAs with the condition K<1.

### 2.3. Modified Cosine Similarity Measure of BPAs

A modified cosine similarity measure is proposed by Jiang [42]. Because it considers three important factors, namely, angle, distance, and vector norm, the modified cosine similarity measure is an efficient approach to measure the similarity between vectors more precisely. The modified cosine similarity measure among the BPAs can determine whether the pieces of evidence conflict with each other. A large similarity indicates that this piece of evidence has more support from another piece of evidence, while a small similarity indicates that this piece of evidence has less support from another piece of evidence.

**Definition** **5.** *Let $E=[e_{1},e_{2},\ldots ,e_{n}]$ and $F=[f_{1},f_{2},\ldots ,f_{n}]$ be two vectors of $R^{n}$. The modified cosine similarity between vectors E and F is defined as*

(9) $$SI(E,F)=\left\{\begin{array}{ll} \frac{1}{2}\left\{\alpha^{-P}+min\left(\frac{\left|E\right|}{\left|F\right|},\frac{\left|F\right|}{\left|E\right|}\right)\right\}si_{cos}\left(E,F\right), & E\neq 0,F\neq 0,\\ 0, & E=0\;or\;F=0, \end{array}\right.$$

*where α is a constant whose value is greater than 1, P is the Euclidean distance between the two vectors E and F, $\alpha^{-P}$ is the distance-based similarity measure, $min(\frac{\left|E\right|}{\left|F\right|},\frac{\left|F\right|}{\left|E\right|})$ is the minimum of $\frac{\left|E\right|}{\left|F\right|}$ and $\frac{\left|F\right|}{\left|E\right|}$, and $si_{cos}\left(E,F\right)$ is the cosine similarity. The larger the α is, the greater the distance impact on vector similarity will be.*

**Definition** **6.** *Let $m_{1}$ and $m_{2}$ be the BPAs in the frame of discernment $U=\{C_{1},C_{2},\ldots ,C_{N}\}$. The two vectors are expressed as*

(10) $$\begin{array}{cc} Bel_{i} & =[Bel_{i}\left(C_{1}\right),Bel_{i}\left(C_{2}\right),\ldots ,Bel_{i}\left(C_{N}\right)],\;i=1,2,\\ Pl_{i} & =[Pl_{i}\left(C_{1}\right),Pl_{i}\left(C_{2}\right),\ldots ,Pl_{i}\left(C_{N}\right)],\;i=1,2. \end{array}$$

*Then, the belief function vector similarity $SI(Bel_{1},Bel_{2})$ and the plausibility function vector similarity $SI(Pl_{1},Pl_{2})$ can be calculated. The new similarity of BPAs is defined as*

(11) $$SI_{BPA}=\left(1-\lambda \right)*SI\left(Bel_{1},Bel_{2}\right)+\lambda *SI\left(Pl_{1},Pl_{2}\right),$$

*with*

(12) 0≤λ≤1,

*where λ is the total uncertainty of BPAs, which is defined as*

(13) $$\lambda =\frac{\sum_{i=1}^{2}\sum_{j=1}^{N}\left(Pl_{i}\left(C_{j}\right)-Bel_{i}\left(C_{j}\right)\right)}{\sum_{i=1}^{2}\sum_{j=1}^{N}\left(Pl_{i}\left(C_{j}\right)\right)}.$$

Because $Pl_{i}\left(C_{j}\right)\geq Bel_{i}\left(C_{j}\right)$ and Bel≥0, if $Pl_{i}\left(C_{j}\right)=Bel_{i}\left(C_{j}\right)$, then λ=0. Otherwise, if $Bel_{i}\left(C_{j}\right)=0$, then λ=1. The larger the uncertainty λ is, the greater the influence on the similarity of BPA will be.

### 2.4. Belief Entropy

A novel type of belief entropy, known as the Deng entropy, was first proposed by Deng [43]. When the uncertain information is expressed by probability, the Deng entropy degenerates to the Shannon entropy. Hence, the Deng entropy is regarded as a generalization of the Shannon entropy. It is an efficient mathematical tool to measure the uncertain information, especially when the uncertain information is expressed by the BPA. Because of its advantage in measuring the uncertain information, the Deng entropy is applied in a variety of areas [57,58]. The basic concepts are introduced below.

**Definition** **7.** *Let B be a hypothesis or proposition of the BPA m in the frame of discernment U and |B| be the cardinality of B. The Deng entropy of the BPA m is defined as follows:*

(14) $$E_{d}\left(m\right)=-\sum_{B\subseteq U}m\left(B\right)\;log\;\frac{m(B)}{2^{\left|B\right|}-1}.$$

*When the belief value is only allocated to the singleton, the Deng entropy degenerates to the Shannon entropy, i.e.,*

(15) $$E_{d}\left(m\right)=-\sum_{B\in U}m\left(B\right)\;log\;\frac{m(B)}{2^{\left|B\right|}-1}=-\sum_{B\in U}m\left(B\right)\;log\;m\left(B\right).$$

The larger the value of the cardinality of the hypothesis or proposition, the larger the value the Deng entropy of evidence, which means that the piece of evidence involves more information. Therefore, if a piece of evidence has a large Deng entropy value, it has more support from other pieces of evidence, indicating that this piece of evidence plays an important role in the evidence combination.

## 3. The Proposed Method

In this paper, a weighted combination method for conflicting pieces of evidence multi-sensor data fusion is proposed by combining the modified cosine similarity measure of evidence with the belief entropy function. In contrast to the method of Jiang et al. [42], in the proposed method, the impact of evidence itself is considered in the process of fusion of multiple pieces of evidence by leveraging the belief entropy [43], i.e., a useful uncertainty measure tool, to measure the information volume of each piece of evidence, so that the proposed method can combine multiple pieces of evidence with greater accuracy. This will be discussed further in the next section.

### 3.1. Process Steps

**Figure 1:**
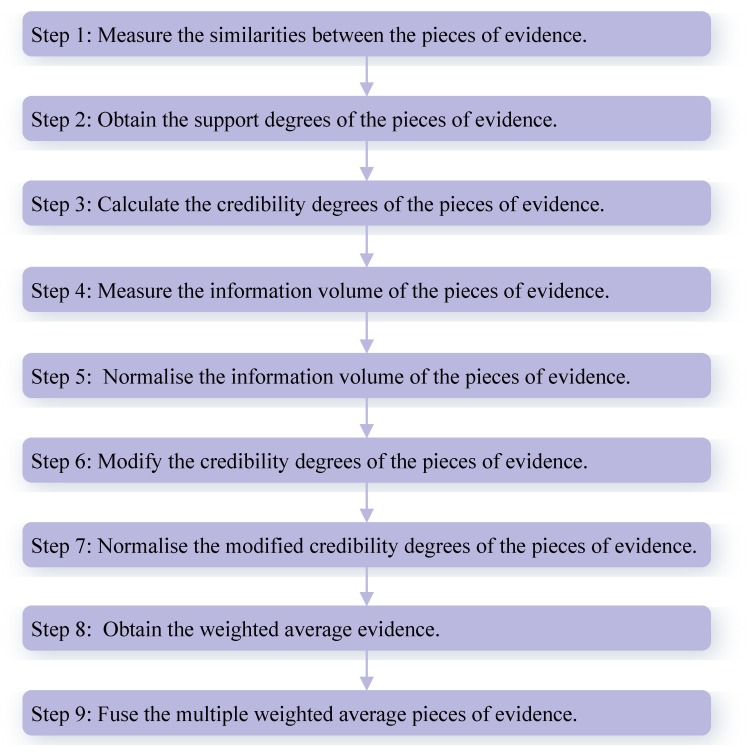
The flowchart of the proposed method.

The proposed method is composed of the following procedures. The credibility degree of the pieces of evidence is first determined on the basis of the similarity measure among the BPAs. Then, the credibility degree is modified by leveraging the belief entropy function to measure the information volume of the evidence. Afterwards, the final weight of each piece of evidence is obtained and adopted to adjust the body of evidence before using Dempster’s combination rule. The specific calculation processes are listed as follows. The flowchart of the proposed method is shown in Figure 1.

Step 1: Measure the similarities between the pieces of evidence.

The similarity measure $SI_{BPA}\left(ij\right)$ between the BPAs $m_{i}$ and $m_{j}$ can be obtained by Equations (11)–(13). Then, a similarity measure matrix (SMM) can be constructed as follows:

(16) $$SMM=\left[\begin{array}{ccccc} SI_{BPA}\left(11\right) & \cdots & SI_{BPA}\left(1i\right) & \cdots & SI_{BPA}\left(1k\right)\\ \vdots & \vdots & \vdots & \vdots & \vdots \\ SI_{BPA}\left(i1\right) & \cdots & SI_{BPA}\left(ii\right) & \cdots & SI_{BPA}\left(ik\right)\\ \vdots & \vdots & \vdots & \vdots & \vdots \\ SI_{BPA}\left(k1\right) & \cdots & SI_{BPA}\left(ki\right) & \cdots & SI_{BPA}\left(kk\right) \end{array}\right].\;$$

Step 2: Obtain the support degrees of the pieces of evidence.

The support degree of the BPA $m_{i}$(i=1,…,k), denoted as $SD(m_{i})$, is defined as follows:

(17) $$SD\left(m_{i}\right)=\sum_{j=1,j\neq i}^{k}SI_{BPA}\left(ij\right).$$

Step 3: Calculate the credibility degrees of the pieces of evidence.

The credibility degree of the BPA $m_{i}$(i=1,…,k), denoted as $CD(m_{i})$, is defined as follows:

(18) $$CD\left(m_{i}\right)=\frac{SD(m_{i})}{\sum_{l=1}^{k}SD\left(m_{l}\right)}.$$

Step 4: Measure the information volume of the pieces of evidence.

According to Equation (14), the belief entropy $E_{d}\left(m_{i}\right)$ of the BPA $m_{i}$(i=1,…,k) can be calculated. To avoid assigning zero weight to the evidence, the information volume $IV(m_{i})$ is used for measuring the uncertain information of $m_{i}$. It is defined as follows:

(19) $$IV\left(m_{i}\right)=e^{E_{d}\left(m_{i}\right)}=e^{-\sum_{B\subseteq U}m\left(B\right)\;log\;\frac{m(B)}{2^{\left|B\right|}-1}}.$$

Step 5: Normalize the information volume of the pieces of evidence.

The information volume of the BPA $m_{i}$(i=1,…,k) will be normalized as below:

(20) $$\bar{IV}\left(m_{i}\right)=\frac{IV(m_{i})}{\sum_{l=1}^{k}IV\left(m_{l}\right)}.$$

Step 6: Modify the credibility degrees of the pieces of evidence.

Based on the normalized information volume, the credibility degree of the BPA $m_{i}$(i=1,…,k) will be modified, denoted as $MCD(m_{i})$:

(21) $$MCD\left(m_{i}\right)=CD\left(m_{i}\right)\times \bar{IV}{\left(m_{i}\right)}^{\left(\frac{\sum_{l=1}^{k}CD\left(m_{l}\right)}{k}-CD\left(m_{i}\right)\right)}.$$

Step 7: Normalize the modified credibility degrees of the pieces of evidence.

The modified credibility degree $MCD(m_{i})$ of the BPA $m_{i}(i=1,\ldots ,k$) will be normalized as below, and is considered as the final weight to adjust the bodies of evidence.

(22) $$\bar{M}CD\left(m_{i}\right)=\frac{MCD(m_{i})}{\sum_{l=1}^{k}MCD\left(m_{l}\right)}.$$

Step 8: Obtain the weighted average evidence.

Based on the modified credibility degree of the BPA $m_{i}$(i=1,…,k), the weighted average evidence WAE(m) is defined as follows:

(23) $$WAE\left(m\right)=\sum_{i=1}^{k}\left(\bar{M}CD\left(m_{i}\right)\times m_{i}\right).$$

Step 9: Fuse multiple weighted average pieces of evidence.

When *k* number of pieces of evidence exist, the weighted average evidence will be fused through Dempster’s combination rule Equation (7) via k−1 times as below,

(24) $$Fus\left(m\right)={\left({\left({\left(WAE\left(m\right)⊕WAE\left(m\right)\right)}_{1}⊕\cdots \right)}_{h}⊕WAE\left(m\right)\right)}_{\left(k-1\right)}.$$

Ultimately, we can obtain the final fusion result of the evidence.

### 3.2. Algorithm

Let $m=\{m_{1},\ldots ,m_{i},\ldots ,m_{k}\}$ be a set of multiple pieces of evidence. After receiving *k* pieces of evidence, a fusion result is expected to be generated for decision-making support. The weighted fusion method for multiple pieces of evidence is outlined in Algorithm 1.

As shown in Algorithm 1, it provides a formal expression in terms of the specific calculation processes of the proposed method listed in Section 3.1. To be specific, Lines 2–7 explain how to measure the similarities between the pieces of evidence and construct the similarity measure matrix for *k* pieces of evidence. Lines 9–11 show how to obtain the support degrees for *k* pieces of evidence. Lines 13–15 represent how to calculate the credibility degrees for *k* pieces of evidence. Lines 17–19 explain how to measure the information volumes for *k* pieces of evidence. Lines 21–23 express how to normalize the information volumes for *k* pieces of evidence. Lines 25–27 state how to modify the credibility degrees for *k* pieces of evidence. Lines 29–31 show how to normalize the modified credibility degrees for *k* pieces of evidence. Line 33 describes how to obtain the weighted average evidence based on *k* pieces of evidence. Lines 35–37 depict how to generate the fusion result.


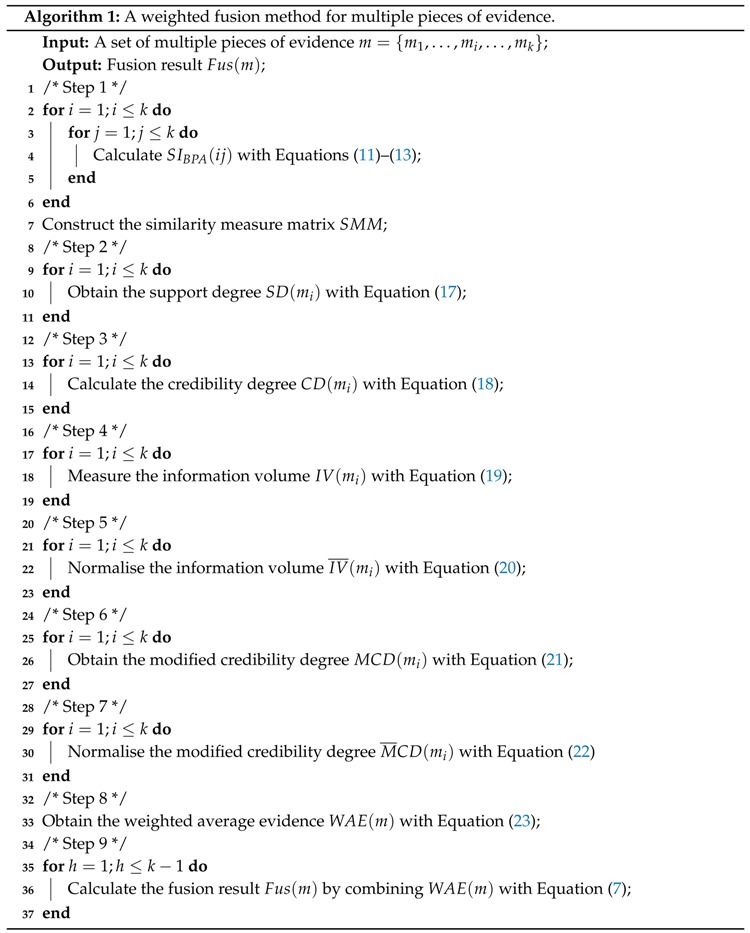


## 4. Numerical Example

In this section, in order to demonstrate the feasibility and effectiveness of the proposed method, a numerical example is illustrated.

**Example** **1.** *Consider the decision-making problem of the multi-sensor-based target recognition system from [59] associated with five different kinds of sensors to observe objects, where U={a,b,c}. Here, a, b, and c are the three objects in the frame of discernment U. The five BPAs that are collected by the system are listed as shown in Table 1.*

Step 1: The similarity measure $SI_{BPA}\left(ij\right)$(i,j=1,2,3,4,5) between the BPAs $m_{i}$ and $m_{j}$ can be constructed as below:
  $$SMM=\left(\begin{array}{ccccc} 1.0000 & 0.3730 & 0.8144 & 0.7478 & 0.7478\\ 0.3730 & 1.0000 & 0.1958 & 0.1568 & 0.1568\\ 0.8144 & 0.1958 & 1.0000 & 0.9340 & 0.9340\\ 0.7478 & 0.1568 & 0.9340 & 1.0000 & 1.0000\\ 0.7478 & 0.1568 & 0.9340 & 1.0000 & 1.0000 \end{array}\right).$$
Step 2: The support degree SD(m) of the BPA $m_{i}$(i=1,2,3,4,5) is calculated as shown in Table 2.
Step 3: The credibility degree CD(m) of the BPA $m_{i}$(i=1,2,3,4,5) is obtained as shown in Table 2.
Step 4: The information volume IV(m) of the BPA $m_{i}$(i=1,2,3,4,5) is measured as shown in Table 2.
Step 5: The information volume of the BPA $m_{i}$(i=1,2,3,4,5) is normalized as shown in Table 2, denoted by $\bar{IV}\left(m\right)$.
Step 6: The credibility degree MCD(m) of the BPA $m_{i}$(i=1,2,3,4,5) is modified as shown in Table 2.
Step 7: The modified credibility degree $\bar{M}CD\left(m\right)$ of the BPA $m_{i}$(i=1,2,3,4,5) is normalized as shown in Table 2.
Step 8: The weighted average evidence WAE(m) is computed as shown in Table 3.
Step 9: By fusing the weighted average evidence via Dempster’s combination rule four times, the final fusion result Fus(m) of evidence can be produced as shown in Table 3.

**Figure 2:**
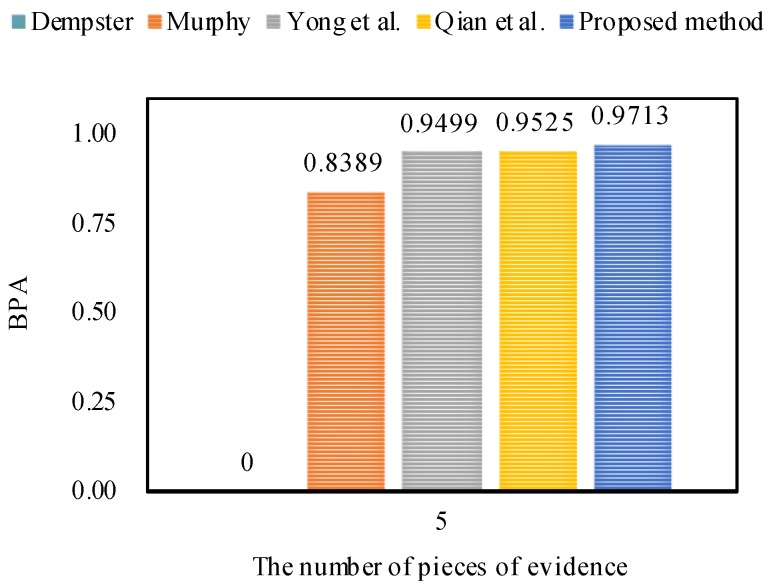
The comparisons of target *a*’s BPA in terms of different methods.

From Example 1, it is obvious that $m_{2}$ highly conflicts with other pieces of evidence. The fusing results that are obtained by different combination approaches are presented in Table 4. In addition, the comparisons of target *a*’s BPA in terms of different combination rules are shown in Figure 2.

As shown in Table 4, no matter how many pieces of evidence support target *a*, Dempster’s combination method [5] always generates a counterintuitive result. As the number of pieces of evidence increases to three, Murphy’s combination method [40] and Deng et al.’s combination method [41] cannot deal with the highly conflicting pieces of evidence very well, because the BPA values of object *a* generated by Murphy’s method [40] and Deng et al.’s method [41] are 33.24% and 44.77%, respectively, which are smaller than 50%. When the number of pieces of evidence increases from four to five, Murphy’s combination method [40] and Deng et al.’s combination method [41] work well, and the BPA values of object *a* generated by Murphy’s method [40] and Deng et al.’s method [41] increase up to 83.89% and 94.99%, respectively.

On the other hand, as shown in Table 4, Qian et al.’s combination method [59] and the proposed method show reasonable results and can efficiently deal with the highly conflicting pieces of evidence as the number of pieces of evidence increases from three to five. In the face of five pieces of evidence, the BPA value of object *a* generated by the proposed method increases to 97.13% which is much higher than for other combination approaches, as shown in Figure 2. Therefore, it is concluded that the proposed method is as feasible and effective as related approaches.

## 5. Statistical Experiment

In this section, in order to make a sound comparison, a statistical experiment is carried out with multiple pieces of initial data for the comparison of the proposed method with other related methods.

This statistical experiment is implemented based on Example 1. In the experimental setting, for generating multiple initial data 100 times, we provide a variation range [−0.1, 0.1] for each BPA of $m_{1}$, and vary the values of BPAs of $m_{1}$ randomly.

Then, the generated multiple pieces of initial data are fused by utilizing the different methods, namely, Dempster’s combination method [5], Murphy’s combination method [40], Deng et al.’s combination method [41], Jiang et al.’s combination method [42], and the proposed method.

**Figure 3:**
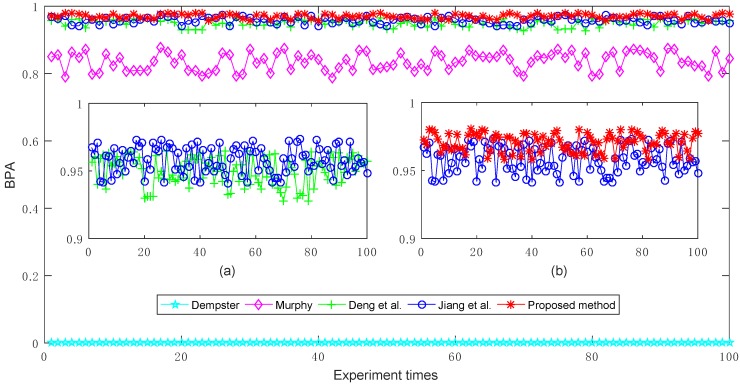
The comparisons of target *a*’s BPAs obtained by different combination methods where the multiple BPAs are generated randomly 100 times. (**a**) The comparisons of Deng et al.’s combination method and Jiang et al.’s combination method; (**b**) The comparisons of Jiang et al.’s combination method and the proposed method.

The experimental results of target *a*’s BPA generated by different combination methods are shown in Figure 3. From the comparison results, it is obvious that Murphy’s combination method [40], Deng et al.’s combination method [41], Jiang et al.’s combination method [42], and the proposed method are more efficient than Dempster’s combination method [5], because Dempster’s combination method cannot effectively deal with the conflicting pieces of evidence, and thus always generates counterintuitive results where target *a*’s BPA value is 0 (under 0.5). In contrast, the other methods can effectively cope with the conflicting evidence and recognize the target *a*, where its corresponding BPA value is always larger than 0.5 under multiple experiments. On the other hand, because Murphy’s combination method is a simply average-weighted approach to the bodies of evidence, its overall performance is poorer than that of Deng et al.’s combination method, Jiang et al.’s combination method, and the proposed method to a certain extent.

Furthermore, as shown in Figure 3a, Jiang et al.’s combination method [42] which is based on the modified cosine similarity measure, is more effective than Deng et al.’s combination method [41] that is based on the Jousselme distance as a whole. This is the reason that the modified cosine similarity measure is considered in this study.

In order to improve the performance of Jiang et al.’s combination method, we investigate and find that in the process of fusion of multiple pieces of evidence, the impact of the evidence itself is overlooked in their method. Hence, we also take the belief entropy into consideration to measure the information volume of each piece of evidence in the course of fusion and design the proposed method. Consequently, as shown in Figure 3b, it can be noted that the proposed method is superior to Jiang et al.’s combination method [42] with a higher target *a* BPA value.

## 6. Applications

In this section, the proposed approach is applied to Iris data set classification and motor rotor fault diagnosis, respectively, to validate its practicability, in which the experimental data in [48,59] are leveraged for the comparison among different approaches.

### 6.1. Iris Data Set Classification

Consider the Iris data set classification problem associated with a frame of discernment *U* consisting of three species of Iris flowers given by *U* = {setosa, versicolor, virginica} = {Se,Ve,Vi} in terms of four numerical attributes of Iris flowers given by {sepallength(SL), sepalwidth(SW), petallength(PL), petalwidth(PW)}, where the BPAs of Iris instances are modeled with noisy data and given in Table 5 from [59].

Step 1: The similarity measure $SI_{BPA}\left(ij\right)$(i,j=SL,SW,PL,PW) between the BPAs $m_{i}$ and $m_{j}$ can be constructed as below:
  $$SMM=\left(\begin{array}{cccc} 1.0000 & 0.3324 & 0.7965 & 0.7750\\ 0.3324 & 1.0000 & 0.2056 & 0.1794\\ 0.7965 & 0.2056 & 1.0000 & 0.9867\\ 0.7750 & 0.1794 & 0.9867 & 1.0000 \end{array}\right).$$
Step 2: The support degree of the BPA $m_{i}\left(i=SL,SW,PL,PW\right)$ is calculated as follows:
  $$\begin{array}{c} SD(m_{SL})=1.9039,\\ SD(m_{SW})=0.7174,\\ SD(m_{PL})=1.9888,\\ SD(m_{PW})=1.9411. \end{array}$$
Step 3: The credibility degree of the BPA $m_{i}\left(i=SL,SW,PL,PW\right)$ is obtained as below:
  $$\begin{array}{c} CD(m_{SL})=0.2906,\\ CD(m_{SW})=0.1095,\\ CD(m_{PL})=0.3036,\\ CD(m_{PW})=0.2963. \end{array}$$
Step 4: The information volume of the BPA $m_{i}\left(i=SL,SW,PL,PW\right)$ is measured as follows:
  $$\begin{array}{c} IV(m_{SL})=7.8287,\\ IV(m_{SW})=1.0842,\\ IV(m_{PL})=3.4202,\\ IV(m_{PW})=3.4998. \end{array}$$
Step 5: The information volume of the BPA $m_{i}\left(i=SL,SW,PL,PW\right)$ is normalised as follows:
  $$\begin{array}{c} \bar{IV}\left(m_{SL}\right)=0.4945,\\ \bar{IV}\left(m_{SW}\right)=0.0685,\\ \bar{IV}\left(m_{PL}\right)=0.2160,\\ \bar{IV}\left(m_{PW}\right)=0.2210. \end{array}$$
Step 6: The credibility degree of the BPA $m_{i}\left(i=SL,SW,PL,PW\right)$ is modified as below:
  $$\begin{array}{c} MCD(m_{SL})=0.2991,\\ MCD(m_{SW})=0.0751,\\ MCD(m_{PL})=0.3296,\\ MCD(m_{PW})=0.3177. \end{array}$$
Step 7: The modified credibility degree of the BPA $m_{i}\left(i=SL,SW,PL,PW\right)$ is normalized as follows:
  $$\begin{array}{c} \bar{M}CD\left(m_{SL}\right)=0.2928,\\ \bar{M}CD\left(m_{SW}\right)=0.0736,\\ \bar{M}CD\left(m_{PL}\right)=0.3226,\\ \bar{M}CD\left(m_{PW}\right)=0.3111. \end{array}$$
Step 8: The weighted average evidence is computed as below:
  $$\begin{array}{l} m(\{Se\})=0.5314,\\ m(\{Ve\})=0.3080,\\ m(\{Vi\})=0.1322,\\ m(\{Se,Ve\})=0.0090,\\ m(\{Se,Vi\})=0.0015,\\ m(\{Ve,Vi\})=0.0164,\\ m(\{Se,Ve,Vi\})=0.0015. \end{array}$$
Step 9: By fusing the weighted average evidence via Dempster’s combination rule four times, the final fusion result of the evidence can be produced as follows:
  $$\begin{array}{l} m(\{Se\})=0.8693,\\ m(\{Ve\})=0.1254,\\ m(\{Vi\})=0.0053,\\ m(\{Se,Ve\})=1\times 10^{-7},\\ m(\{Se,Vi\})=7\times 10^{-10},\\ m(\{Ve,Vi\})=1\times 10^{-6},\\ m(\{Se,Ve,Vi\})=5\times 10^{-11}. \end{array}$$

The fusion results based on different combination approaches that were applied on the Iris data set are presented in Table 6. From the experimental results, it can be seen that Dempster’s combination method [5] and Murphy’s combination method [40] always generate counterintuitive results and classify the species of Iris flower as versicolor, even when the number of pieces of evidence increases from two ($m_{SL},m_{SW}$) to four ($m_{SL},m_{SW},m_{PL},m_{PW}$). By contrast, Deng et al.’s combination method [41] works well when the number of pieces of evidence is increased up to four ($m_{SL},m_{SW},m_{PL},m_{PW}$), because it can classify the species of Iris flower as the target setosa with a belief value of 73.01%.

**Figure 4:**
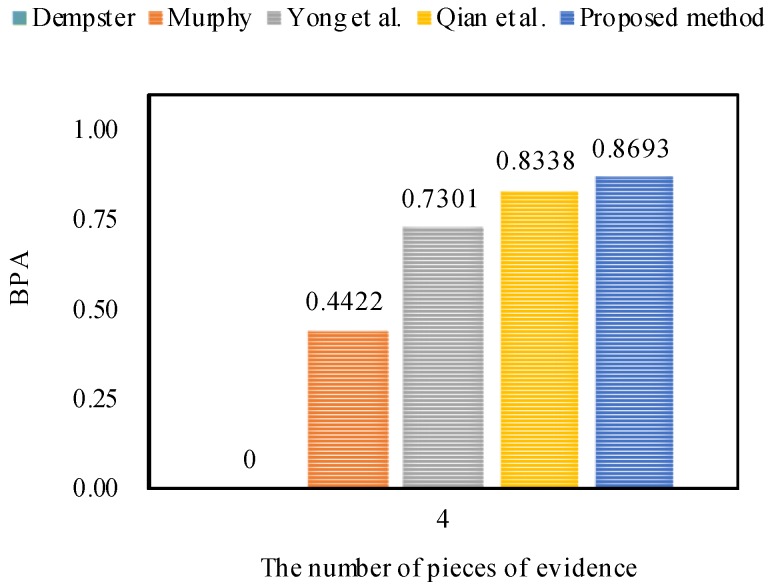
The comparisons of target Se’s BPA in terms of different methods.

Obviously, Qian et al.’s combination method [59] and the proposed method show reasonable results and classify the species of Iris flower as the target setosa with 83.38% and 86.93% belief values, respectively. Therefore, we can conclude that the proposed method is more efficient than other related methods with better accuracy of data classification, as shown in Figure 4. The reason is that the proposed method not only takes the interplay between the pieces of evidence into account, but also considers the impacts of the pieces of evidence themselves.

### 6.2. Motor Rotor Fault Diagnosis

Supposing there are three types of faults for a motor rotor given by $\left\{F_{1},F_{2},F_{3}\right\}$ = {rotorunbalance, rotormisalignment, pedestallooseness} in the frame of discernment *U*. We place a set of vibration acceleration sensors at different places for gathering the vibration signals given by *S* = $\left\{S_{1},S_{2},S_{3}\right\}$. The acceleration vibration frequency amplitudes at 1X, 2X, and 3X frequencies are considered as the fault feature variables. The collected sensor reports at 1X, 2X, and 3X frequencies modeled as BPAs are shown in Table 7, Table 8 and Table 9, respectively, in which $m_{1}\left(\cdot \right)$, $m_{2}\left(\cdot \right)$, and $m_{3}\left(\cdot \right)$ represent the BPAs modeled from the three vibration acceleration sensors $S_{1}$, $S_{2}$, and $S_{3}$.

#### 6.2.1. Motor Rotor Fault Diagnosis at 1X Frequency

By conducting the steps in Section 3, the weighted average evidence with regard to motor rotor fault diagnosis at 1X frequency is obtained as below:

$$\begin{array}{c} m(\left\{F_{2}\right\})=0.5442,\\ m(\left\{F_{3}\right\})=0.0006,\\ m(\left\{F_{1},F_{2}\right\})=0.0773,\\ m(\left\{F_{1},F_{2},F_{3}\right\})=0.3780. \end{array}$$

Then, the final fusion results for motor rotor fault diagnosis at 1X frequency are computed as follows:

$$\begin{array}{c} m(\left\{F_{2}\right\})=0.9055,\\ m(\left\{F_{3}\right\})=0.0002,\\ m(\left\{F_{1},F_{2}\right\})=0.0404,\\ m(\left\{F_{1},F_{2},F_{3}\right\})=0.0541. \end{array}$$

#### 6.2.2. Motor Rotor Fault Diagnosis at 2X Frequency

By carrying out the steps in Section 3, the weighted average evidence with respect to motor rotor fault diagnosis at 2X frequency is obtained as follows:

$$\begin{array}{c} m(\left\{F_{2}\right\})=0.7387,\\ m(\left\{F_{1},F_{2},F_{3}\right\})=0.2613. \end{array}$$

Afterwards, the final fusion results in terms of motor rotor fault diagnosis at 2X frequency are generated as below:

$$\begin{array}{c} m(\left\{F_{2}\right\})=0.9822,\\ m(\left\{F_{1},F_{2},F_{3}\right\})=0.0178. \end{array}$$

#### 6.2.3. Motor Rotor Fault Diagnosis at 3X Frequency

By applying the steps in Section 3, the weighted average evidence with respect to motor rotor fault diagnosis at 3X frequency is obtained as follows:

$$\begin{array}{c} m(\left\{F_{1}\right\})=0.3111,\\ m(\left\{F_{2}\right\})=0.4346,\\ m(\left\{F_{1},F_{2}\right\})=0.2115,\\ m(\left\{F_{1},F_{2},F_{3}\right\})=0.0428. \end{array}$$

Then, the final combination results for motor rotor fault diagnosis at 3X frequency are shown below:

$$\begin{array}{c} m(\left\{F_{1}\right\})=0.3345,\\ m(\left\{F_{2}\right\})=0.6321,\\ m(\left\{F_{1},F_{2}\right\})0.0333,\\ m(\left\{F_{1},F_{2},F_{3}\right\})=0.0001. \end{array}$$

From the experimental results as shown in Table 10, Table 11 and Table 12, it can be seen that the proposed method diagnoses the fault type as $F_{2}$, in accordance with Jiang et al.’s method [48].

**Figure 5:**
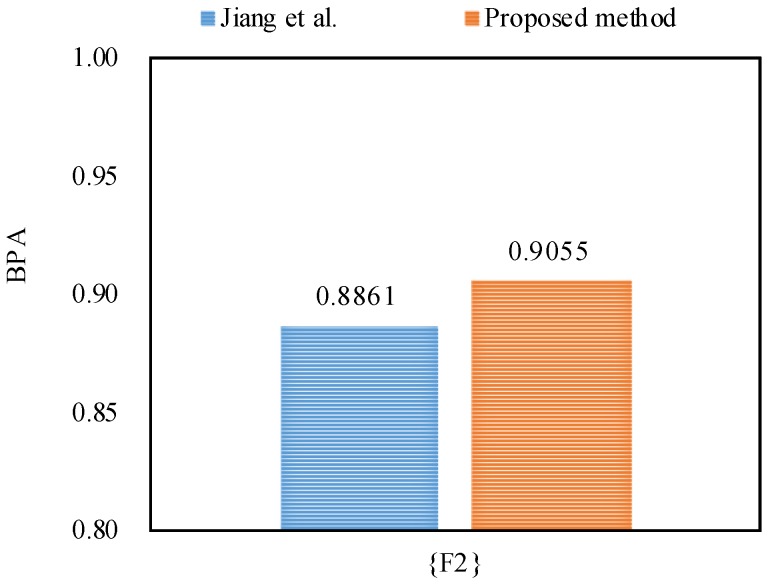
The comparison of the BPA of the target $F_{2}$ at 1X frequency.

**Figure 6:**
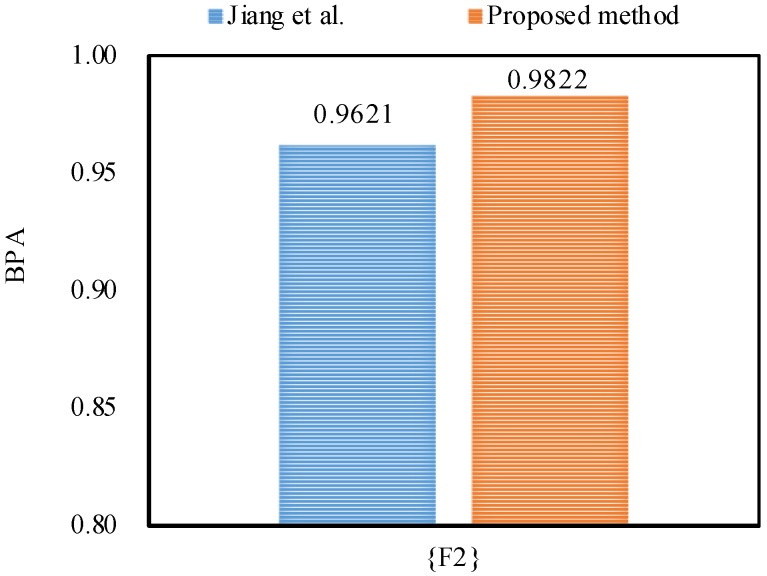
The comparison of the BPA of the target $F_{2}$ at 2X frequency.

**Figure 7:**
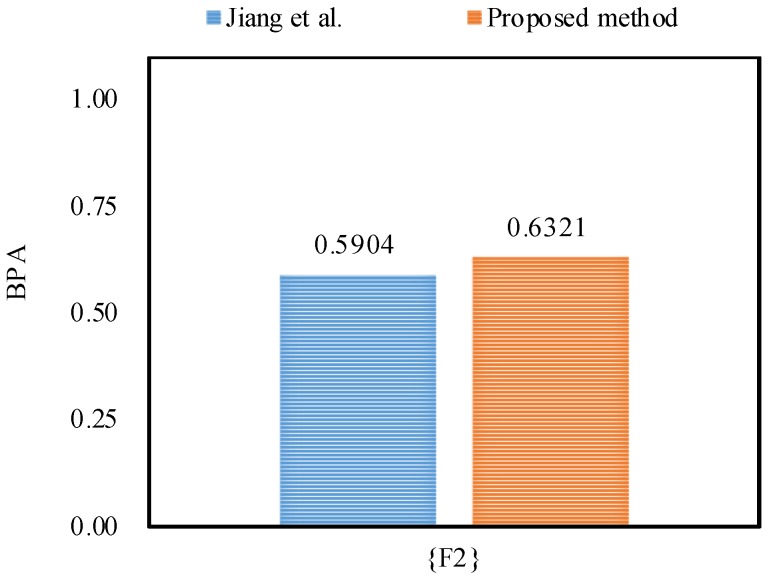
The comparison of the BPA of the target $F_{2}$ at 3X frequency.

Furthermore, the proposed method outperforms Jiang et al.’s method [48] in dealing with the uncertainty as shown in Figure 5, Figure 6 and Figure 7, because by utilizing the proposed method, the belief degrees allocated to the target fault type $F_{2}$ at 1X frequency, 2X frequency and 3X frequency increase up to 90.55%, 98.22%, and 63.21%, respectively; however, by using Jiang et al.’s method [48], the belief degrees allocated to the target $F_{2}$ at 1X frequency, 2X frequency and 3X frequency are 88.61%, 96.21%, and 59.04%, respectively.

Additionally, by utilizing the proposed method, the uncertainty $\left\{F_{1},F_{2}\right\}$ falls from 0.0582 to 0.0541, and the uncertainty $\left\{F_{1},F_{2},F_{3}\right\}$ falls from 0.0555 to 0.0404 at 1X frequency; the uncertainty $\left\{F_{1},F_{2},F_{3}\right\}$ decreased from 0.0371 to 0.0178 at 2X frequency; the uncertainty $\left\{F_{1},F_{2}\right\}$ falls from 0.0651 to 0.0333, and the uncertainty $\left\{F_{1},F_{2},F_{3}\right\}$ drops from 0.0061 to 0.0001 at 3X frequency. As a result, the proposed method can diagnose motor rotor faults more accurately than the related work.

## 7. Conclusions

In this paper, a weighted combination method for conflicting evidence in multi-sensor data fusion was proposed by combining the modified cosine similarity measure of the pieces of evidence with the belief entropy function. The proposed method was a kind of pretreatment of the bodies of evidence, which was effective to handle the conflicting pieces of evidence in a multi-sensor environment. A numerical example was illustrated to show the feasibility and effectiveness of the proposal. In addition, applications in data classification and motor rotor fault diagnosis were presented to validate the practicability of the proposed method, where it outperformed the related methods with better accuracy.

## Figures and Tables

**Table 1:** The basic probability assignments (BPAs) for the example.

Pieces of Evidence	BPAs			
	{a}	{b}	{c}	{a,b,c}
$m_{1}\left(\cdot \right)$	0.30	0.20	0.10	0.40
$m_{2}\left(\cdot \right)$	0.00	0.90	0.10	0.00
$m_{3}\left(\cdot \right)$	0.60	0.10	0.10	0.20
$m_{4}\left(\cdot \right)$	0.70	0.10	0.10	0.10
$m_{5}\left(\cdot \right)$	0.70	0.10	0.10	0.10

**Table 2:** The calculated results in terms of support degree, credibility degree, information volume, normalized information volume, credibility degree, and modified credibility degree of BPAs.

Items	Pieces of Evidence				
	*m*_1_	*m*_2_	*m*_3_	*m*_4_	*m*_5_
*SD*(*m*)	2.6830	0.8824	2.8782	2.8386	2.8386
*CD*(*m*)	0.2214	0.0728	0.2375	0.2342	0.2342
*IV*(*m*)	19.480	1.5984	8.4351	5.1423	5.1423
*IV*(*m*)	0.4895	0.0402	0.2119	0.1292	0.1292
*MCD*(*m*)	0.2248	0.0484	0.2517	0.2512	0.2512
*M**CD*(*m*)	0.2188	0.0471	0.2450	0.2445	0.2445

**Table 3:** The weighted average evidence (*WAE*(*m*)) and final fusion result (*Fus*(*m*)).

Items	BPAs			
	{a}	{b}	{c}	{a,b,c}
*WAE*(*m*)	0.5550	0.1596	0.1000	0.1854
*Fus*(*m*)	0.9713	0.0204	0.0073	0.0010

**Table 4:** Evidence fusion results based on different combination rules.

Evidences	Methods	BPAs				Target
		{a}	{b}	{c}	{a,b,c}	
$m_{1},m_{2}$	Dempster [5]	0.0000	0.9153	0.0847	0.0000	b
	Murphy [40]	0.1187	0.7518	0.0719	0.0576	b
	Deng et al. [41]	0.1187	0.7518	0.0719	0.0576	b
	Qian et al. [59]	0.1187	0.7518	0.0719	0.0576	b
	Proposed method	0.1187	0.7518	0.0719	0.0576	b
$m_{1},m_{2},m_{3}$	Dempster [5]	0.0000	0.9153	0.0847	0.0000	b
	Murphy [40]	0.3324	0.5909	0.0540	0.0227	b
	Deng et al. [41]	0.4477	0.4546	0.0644	0.0333	-
	Qian et al. [59]	0.6110	0.2861	0.0659	0.0370	a
	Proposed method	0.5779	0.3070	0.0714	0.0438	a
$m_{1},m_{2},m_{3},m_{4}$	Dempster [5]	0.0000	0.9153	0.0847	0.0000	b
	Murphy [40]	0.6170	0.3505	0.0272	0.0053	a
	Deng et al. [41]	0.8007	0.1640	0.0283	0.0070	a
	Qian et al. [59]	0.8472	0.1221	0.0249	0.0058	a
	Proposed method	0.8785	0.0857	0.0271	0.0076	a
$m_{1},m_{2},m_{3},m_{4},m_{5}$	Dempster [5]	0.0000	0.9153	0.0847	0.0000	b
	Murphy [40]	0.8389	0.1502	0.0099	0.0010	a
	Deng et al. [41]	0.9499	0.0411	0.0080	0.0010	a
	Qian et al. [59]	0.9525	0.0393	0.0074	0.0008	a
	Proposed method	0.9713	0.0204	0.0073	0.0010	a

**Table 5:** The BPAs of Iris flower instances.

BPAs	Attributes			
	{SL}	{SW}	{PL}	{PW}
m{Se}	0.3337	0.0000	0.6699	0.6996
m{Ve}	0.3165	0.9900	0.2374	0.2120
m{Vi}	0.2816	0.0100	0.0884	0.0658
m{Se,Ve}	0.0307	0.0000	0.0000	0.0000
m{Se,Vi}	0.0052	0.0000	0.0000	0.0000
m{Ve,Vi}	0.0272	0.0000	0.0043	0.0226
m{Se,Ve,Vi}	0.0052	0.0000	0.0000	0.0000

**Table 6:** The comparison of different methods applied in the Iris data set classification.

Evidence	Methods	BPAs							Target
		{Se}	{Ve}	{Vi}	{Se,Ve}	{Se,Vi}	{Ve,Vi}	{Se,Ve,Vi}	
$m_{SL},m_{SW}$	Dempster [5]	0.0000	0.9916	0.0084	0.0000	0.0000	0.0000	0.0000	Ve
	Murphy [40]	0.0655	0.8828	0.0505	$6\times 10^{-4}$	$4\times 10^{-5}$	$5\times 10^{-4}$	$1\times 10^{-5}$	Ve
	Deng et al. [41]	0.0655	0.8828	0.0505	$6\times 10^{-4}$	$4\times 10^{-5}$	$5\times 10^{-4}$	$1\times 10^{-5}$	Ve
	Qian et al. [59]	0.0655	0.8828	0.0505	$6\times 10^{-4}$	$4\times 10^{-5}$	$5\times 10^{-4}$	$1\times 10^{-5}$	Ve
	Proposed method	0.0655	0.8828	0.0505	$6\times 10^{-4}$	$4\times 10^{-5}$	$5\times 10^{-4}$	$1\times 10^{-5}$	Ve
$m_{SL},m_{SW},m_{PL}$	Dempster [5]	0.0000	0.9968	0.0032	0.0000	0.0000	0.0000	0.0000	Ve
	Murphy [40]	0.2112	0.7749	0.0139	$8\times 10^{-6}$	$2\times 10^{-7}$	$9\times 10^{-6}$	$3\times 10^{-8}$	Ve
	Deng et al. [41]	0.3219	0.6534	0.0247	$2\times 10^{-5}$	$4\times 10^{-7}$	$2\times 10^{-5}$	$5\times 10^{-8}$	Ve
	Qian et al. [59]	0.5678	0.4036	0.0287	$2\times 10^{-5}$	$4\times 10^{-7}$	$2\times 10^{-5}$	$5\times 10^{-8}$	Se
	Proposed method	0.5206	0.4421	0.0372	$2\times 10^{-5}$	$5\times 10^{-7}$	$2\times 10^{-5}$	$7\times 10^{-8}$	Se
$m_{SL},m_{SW},m_{PL},m_{PW}$	Dempster [5]	0.0000	0.9988	0.0012	0.0000	0.0000	0.0000	0.0000	Ve
	Murphy [40]	0.4422	0.5546	0.0032	$8\times 10^{-8}$	$5\times 10^{-10}$	$6\times 10^{-7}$	$3\times 10^{-11}$	Ve
	Deng et al. [41]	0.7301	0.2652	0.0047	$1\times 10^{-7}$	$7\times 10^{-10}$	$9\times 10^{-7}$	$5\times 10^{-11}$	Se
	Qian et al. [59]	0.8338	0.1617	0.0045	$9\times 10^{-8}$	$6\times 10^{-10}$	$9\times 10^{-7}$	$4\times 10^{-11}$	Se
	Proposed method	0.8693	0.1254	0.0053	$1\times 10^{-7}$	$7\times 10^{-10}$	$1\times 10^{-6}$	$5\times 10^{-11}$	Se

**Table 7:** The collected sensor reports at the frequency of 1X modeled as BPAs.

BPA	$\left\{F_{2}\right\}$	$\left\{F_{3}\right\}$	$\left\{F_{1},F_{2}\right\}$	$\left\{F_{1},F_{2},F_{3}\right\}$
$m_{1}\left(\cdot \right)$	0.8176	0.0003	0.1553	0.0268
$m_{2}\left(\cdot \right)$	0.5658	0.0009	0.0646	0.3687
$m_{3}\left(\cdot \right)$	0.2403	0.0004	0.0141	0.7452

**Table 8:** The collected sensor reports at the frequency of 2X modeled as BPAs.

BPA	$\left\{F_{2}\right\}$	$\left\{F_{1},F_{2},F_{3}\right\}$
$m_{1}\left(\cdot \right)$	0.6229	0.3771
$m_{2}\left(\cdot \right)$	0.7660	0.2341
$m_{3}\left(\cdot \right)$	0.8598	0.1402

**Table 9:** The collected sensor reports at the frequency of 3X modeled as BPAs.

BPA	$\left\{F_{1}\right\}$	$\left\{F_{2}\right\}$	$\left\{F_{1},F_{2}\right\}$	$\left\{F_{1},F_{2},F_{3}\right\}$
$m_{1}\left(\cdot \right)$	0.3666	0.4563	0.1185	0.0586
$m_{2}\left(\cdot \right)$	0.2793	0.4151	0.2652	0.0404
$m_{3}\left(\cdot \right)$	0.2897	0.4331	0.2470	0.0302

**Table 10:** Fusion results by using different combination methods at 1X frequency.

Method	$\left\{F_{2}\right\}$	$\left\{F_{3}\right\}$	$\left\{F_{1},F_{2}\right\}$	$\left\{F_{1},F_{2},F_{3}\right\}$	Target
Jiang et al. [48]	0.8861	0.0002	0.0582	0.0555	$F_{2}$
Proposed method	0.9055	0.0002	0.0404	0.0541	$F_{2}$

**Table 11:** Fusion results by using different combination methods at 2X frequency.

Method	$\left\{F_{2}\right\}$	$\left\{F_{1},F_{2},F_{3}\right\}$	Target
Jiang et al. [48]	0.9621	0.0371	$F_{2}$
Proposed method	0.9822	0.0178	$F_{2}$

**Table 12:** Fusion results by using different combination methods at 3X frequency.

Method	$\left\{F_{1}\right\}$	$\left\{F_{2}\right\}$	$\left\{F_{1},F_{2}\right\}$	$\left\{F_{1},F_{2},F_{3}\right\}$	Target
Jiang et al. [48]	0.3384	0.5904	0.0651	0.0061	$F_{2}$
Proposed method	0.3345	0.6321	0.0333	0.0001	$F_{2}$
